# Intranasal Esketamine administration in catatonia: a case report.

**DOI:** 10.1192/j.eurpsy.2023.2028

**Published:** 2023-07-19

**Authors:** J. Romay, C. Sevilla, P. Hernandez, I. Lastra, G. Isidro, L. Cayon, G. Cortez, O. Anabitarte, P. Ijalba, M. Gomez Revuelta

**Affiliations:** Psiquiatry, Hospital Universitario Marqués de Valdecilla, Santander, Spain

## Abstract

**Introduction:**

Catatonia is a complex psychomotor syndrome that often goes unrecognized and, consequently, untreated. Prompt and correct identification of catatonia allows for highly effective treatment and prevention of possible complications. Benzodiazepines and electroconvulsive therapy (ECT) are the most widely studied treatment methods. However, no uniform treatment method has yet been brought forward and no previous attempts to treat catatonia on a patient suffering concomitant major depressive disorder (MDD) with NMDA receptor antagonists have been documented so far.

**Objectives:**

To describe the unknown and novel management of catatonia and MDD with intranasal esketamine, a NMDA receptor antagonist.

**Methods:**

A 55-year-old woman with a diagnosis of long-standing recurrent major depressive disorder who was admitted to the psychiatric inpatient unit of UniversityHospital Marqués de Valdecilla (Santander, Spain) suffering a complex catatonic, mutative state framed on a severe MDD. Different ineffective therapeutic interventions were deployed during the course of her illness. After failing to improve under conventional pharmacological treatment and ECT, and given the complexity of peripheral venous access on this patient (which disabled the option for iv ketamine use), we decided to initiate compassionate treatment with intranasal esketamine.

**Results:**

Intranasal esketamine was effective in the resolution of patient’s complex catatonic state. Clinical response from catatonia was observed after 6 intranasal esketamine administrations (2-week follow-up), reaching full catatonia and MDD remission after 12 sessions in absence of significant adverse events

**Conclusions:**

Esketamine showed promising effectiveness for the treatment of catatonia in the context of MDD, although further research on this topic is needed.

**Disclosure of Interest:**

None Declared

